# Genomic characteristics of the first five monkeypox virus clade Ib cases in Thailand: August 2024–March 2025

**DOI:** 10.1128/spectrum.00516-26

**Published:** 2026-09-24

**Authors:** Ravee Nitiyanontakij, Sarinee Reawrang, Duangkamon Siludjai, Pornphimon Kaewsawang, Pimphorn Yooma, Ratchana Ponang, Pawita Suwanvattana, Peera Hemarajata, Wittawin Worakitchanon, Supaporn Wacharapluesadee, Tanawut Srisuk, Thidathip Wongsurawat, Piroon Jenjaroenpun, Worarat Kruasuwan, Pongpun Sawatwong, Woradee Lurchachaiwong, R. Suzanne Beard, Kitpong Sunchatawirul

**Affiliations:** 1 Bamrasnaradura Infectious Diseases Institute, Department of Disease Control, Ministry of Public Health, Nonthaburi, Thailand; 2 Division of Global Health Protection, Thailand Ministry of Public Health – US Centers for Disease Control and Prevention Collaboration, Nonthaburi, Thailand; 3 Association of Public Health Laboratories 8282, Bethesda, Maryland, USA; 4 Emerging Infectious Diseases Clinical Center, Faculty of Medicine, Chulalongkorn University 26683, Bangkok, Thailand; 5 Oxford Nanopore Centre of Excellence, Faculty of Medicine Siriraj Hospital, Mahidol University 65106, Bangkok, Thailand; Arizona State University College of Health Solutions, Phoenix, Arizona, USA

**Keywords:** mpox virus clade Ib, next-generation sequencing, whole-genome sequencing, Thailand, viral evolution

## Abstract

Following the emergence of monkeypox virus clade Ib in the Democratic Republic of the Congo in August 2024, the World Health Organization declared a public health emergency of international concern because of sustained transmission and international spread. We characterized the first clade Ib cases detected in Thailand between August 2024 and March 2025 and assessed their phylogenetic relationships. Whole-genome sequencing was performed from lesion swabs of suspected cases. Phylogenetic analysis, incorporating clade Ia (DQ011155.1) and global clade Ib sequences from GISAID, confirmed all Thai isolates as clade Ib. The genomes showed close relatedness to clade Ib viruses reported internationally, consistent with patients’ travel histories. All isolates exhibited a 1,140-bp deletion in the *C3L* gene region and 52–61 amino acid substitutions compared with the clade Ia reference. Genome sequences were submitted to GISAID and NCBI to support outbreak response and improve understanding of epidemiologic patterns, enabling more effective public health interventions.

**IMPORTANCE:**

On 14 August 2024, the World Health Organization declared the monkeypox virus outbreak in Africa a public health emergency of international concern following the emergence of clade Ib. One week later, Thailand confirmed the first monkeypox clade Ib case in Asia. Through whole-genome sequencing and phylogenomic analysis of five cases detected between August 2024 and March 2025, we identified a 1,140-bp deletion in the *C3L* gene region that distinguishes clade Ib from clade Ia. This deletion directly affects the performance of some commercial real-time PCR assays designed for clade Ia, which rely on amplification of the missing genomic region. Our findings highlight important diagnostic limitations that could lead to misinterpretation or reduced sensitivity in routine testing. Beyond confirming regional introduction linked to global spread, this study underscores the critical role of genomic surveillance in ensuring accurate diagnosis, monitoring viral evolution, and strengthening outbreak response as monkeypox continues to expand internationally.

## INTRODUCTION

Monkeypox virus (MPXV) is a member of the *Orthopoxvirus* genus, encompassing several closely related viruses, including variola, the causative agent of smallpox, and cowpox. The clinical manifestations of monkeypox (MPX) include fever, headache, muscle aches, backache, lymphadenopathy, and a characteristic rash that progresses from macules to papules, vesicles, and pustules (1). The virus is primarily transmitted to humans through direct contact with infected animals, such as rodents and primates, or human-to-human transmission (2). MPXV was found to be transmitted primarily through animal-to-human contact in endemic regions of Central and West Africa (3). In July 2022, the World Health Organization (WHO) declared a large MPX outbreak a public health emergency of international concern due to the surge in cases and the spread of the virus to areas that had previously only seen sporadic infections (3, 4).

The virus has garnered significant attention in recent years due to the emergence of new outbreaks in both endemic and non-endemic regions, leading to a surge of research efforts to understand its genomic characteristics and the distinctions between the various viral clades (5, 6). The MPXV virion consists of an encapsulated double-stranded DNA genome, approximately 197 kbp in length, which encodes approximately 200 putative genes. Inverted terminal repeats (ITR) of 7 kb are located at both ends of the genome. The first clade, formally known as the Central African or Congo Basin (clade I), is generally more virulent and has a higher morbidity and mortality rate compared to the second clade, the West African clade (clade II) (7, 8). Recent outbreaks of MPX have involved both clades, with the 2022 multi-country outbreak primarily linked to the clade II (1, 6).

In August 2024, the WHO declared the MPX outbreak in Africa a public health emergency of international concern due to the emergence of clade Ib (9). The situation of clade Ib has been of particular interest, as this clade has been identified as the predominant cause of recent outbreaks in the Democratic Republic of the Congo, where it has exhibited a pattern of sustained human-to-human transmission in contrast to the more typical zoonotic spillover events associated with the clade Ia lineage in the region (1, 4, 10). Moreover, clade I had not previously been observed outside Central and West Africa, raising concerns about its potential to spread more widely and cause a significant global health impact (6).

Since August 2024, MPX cases linked to the clade Ib variant have been reported in several countries outside of Africa, including Sweden, Thailand, the United Kingdom, California, Germany, India, and China, and were primarily associated with travel histories from Africa and other affected regions (10–16). This study is the first to investigate the genomic characteristics of the initial five patients with a confirmed infection of MPXV clade Ib in Thailand. It examined the virus’s genome and its phylogenetic relationship with other strains from different geographical regions aiming to improve understanding of epidemiological patterns and inform effective public health interventions.

## MATERIALS AND METHODS

### Biosafety

Demographic and epidemiological data were collected from the MPX outbreak questionnaire, which is routinely utilized in the outbreak investigation process by the Division of Epidemiology.

### Specimen and data collection

Specimens were collected from individuals presenting with at least one of the following clinical criteria: fever alone or fever accompanied by symptoms such as sore throat, headache, myalgia, back pain, lymphadenopathy, or skin rashes or pustules. Additionally, suspected MPX cases had a relevant epidemiological linkage within the preceding 21 days, including contact that prompted clinical suspicion, travel to areas reporting MPX cases, participation in events or activities with known cases, occupational exposure to travelers from abroad, or contact with rodents or small mammals originating from Africa. Skin lesions were sampled using sterile flocked swabs inserted into the lesions and placed into viral transport media. All collected specimens were transported and processed following biosafety level 2 enhanced protocols to ensure safety and maintain specimen integrity throughout laboratory procedures. Demographic and epidemiological data were gathered from the MPX outbreak questionnaire, completed by clinical staff on the same day as the specimen collection.

### DNA extraction

All specimens from MPX suspected cases, which are defined as individuals presenting with skin lesions accompanied by systemic symptoms such as fever and lymphadenopathy, and potential epidemiological exposure were transported and processed following biosafety level 2 enhanced protocols. MPXV DNA was extracted from swab samples collected from lesions suspended in viral transport media. This extraction was performed using a magLEAD 12gc nucleic acid extraction system and the MagDEA Dx SV DNA extraction reagent (Precision System Science, Matsudo, Japan). An initial volume of 400 μL of the viral transport media was used for the extraction process following the manufacturer’s instructions, yielding a final elution volume of 50 μL.

### Real-time PCR testing

Real-time PCR was performed to detect MPXV nucleic acid using two different commercial kits. Samples were analyzed to screen MPXV (*F3L* gene) and identify clade Ia (*C3L* gene) and clade II (*J2L*/*J2R* gene) using the Multiplex Real-time PCR Kit for Monkeypox Virus and Central/West African Clade Typing (Uni-Medica, Shenzhen, China), employing the CFX96 Touch Real-Time PCR Detection System (Bio-Rad, CA, USA). The assay was performed for 45 amplification cycles according to the manufacturer’s instructions. The QIAstat-Dx Viral Vesicular Panel assay (QIAGEN Diagnostics GmbH, Hilden, Germany) is a fully automated molecular test utilizing a cartridge that contains all reagents of nucleic acid extraction, multiplex real-time PCR amplification and detection of orthopoxviruses (*F8L* gene), MPXV clade Ia (*C3L* gene), MPXV clade II (*J2L*/*J2R* gene), four human herpesviruses (HSV-1, HSV-2, HHV-6, and VZV), and enterovirus. We performed the clade Ib-specific real-time PCR to confirm clade Ib (dD14–16) using methods reported elsewhere (17). This laboratory-developed assay specifically targets the unique 1,140-bp deletion region of MPXV clade Ib relative to previously described Clade I genomes, including MPXV Clade I reference genome (GenBank accession number DQ011155).

### Whole-genome sequencing

The MPXV genome was sequenced using Illumina and/or Oxford Nanopore Technologies (ONT) platforms. For Illumina sequencing, viral genomes were enriched using the Viral Surveillance Panel V1 (Illumina, CA, USA) library preparation protocol, and libraries were sequenced on the Illumina MiniSeq platform (Illumina, CA, USA). FASTQ files generated by the instrument were used for downstream analyses.

For ONT sequencing, libraries were prepared with the Sequencing Ligation Kit V14 (SQK-LSK114; Oxford Nanopore Technologies, Oxford, UK) according to the manufacturer’s instructions, without enrichment. Libraries were diluted and loaded onto R10.4.1 flow cells (Oxford Nanopore Technologies, Oxford, UK) and sequenced individually per sample on the MinION Mk1B DNA/RNA Sequencing Device with adaptive sampling enabled. Raw POD5 files were basecalled with Dorado (v1.1.1) in SUP mode to generate FASTQ files for downstream analysis.

### Bioinformatics analyses

Different bioinformatics workflows were applied depending on sequencing data availability. For the first case, only Illumina short-read data generated with the Viral Surveillance Panel (VSP) Kit were available. A consensus sequence was obtained using the DRAGEN Microbial Enrichment Plus app (v1.1.1) on BaseSpace (Illumina, CA, USA).

For the remaining four cases, reference-guided assembly was performed using both Illumina and ONT sequencing data. Illumina and ONT FASTQ files for each sample were independently mapped to the MPXV Clade I reference genome (GenBank accession number DQ011155.1) using minimap2 (v2.28), producing one BAM file per platform. Read groups were assigned according to the sequencing platform, and BAM files from the same sample were merged using samtools (v1.20). Variant calling was performed in haploid mode with bcftools (v1.20). BED files were generated to identify low-coverage regions, and consensus sequences were constructed with bcftools (v1.20), incorporating variant calls and masking low-coverage regions, including the 3′ inverted terminal repeat of the MPXV genome. Because Cases 2–5 were assembled from merged Illumina and ONT BAM files, the coverage and depth values reported in Table 2 reflect the combined hybrid alignment for these cases. All five assembled genomes were near-complete (99.1%–100% reference coverage at 966–3,640 × mean depth) and contained the full complement of expected MPXV coding sequences, with the sole structural deletion being the clade Ib–defining 1,140 bp deletion in the *C3L* gene region. Nucleotide and amino acid mutation analyses were performed using Nextclade v3.21.2. Analyses were conducted using the Mpox virus (Clade I) data set from Nextstrain, with the reference genome Zaire_1979-005 (GenBank accession number DQ011155.1)

### Phylogenetic analysis

**Fig 1:**
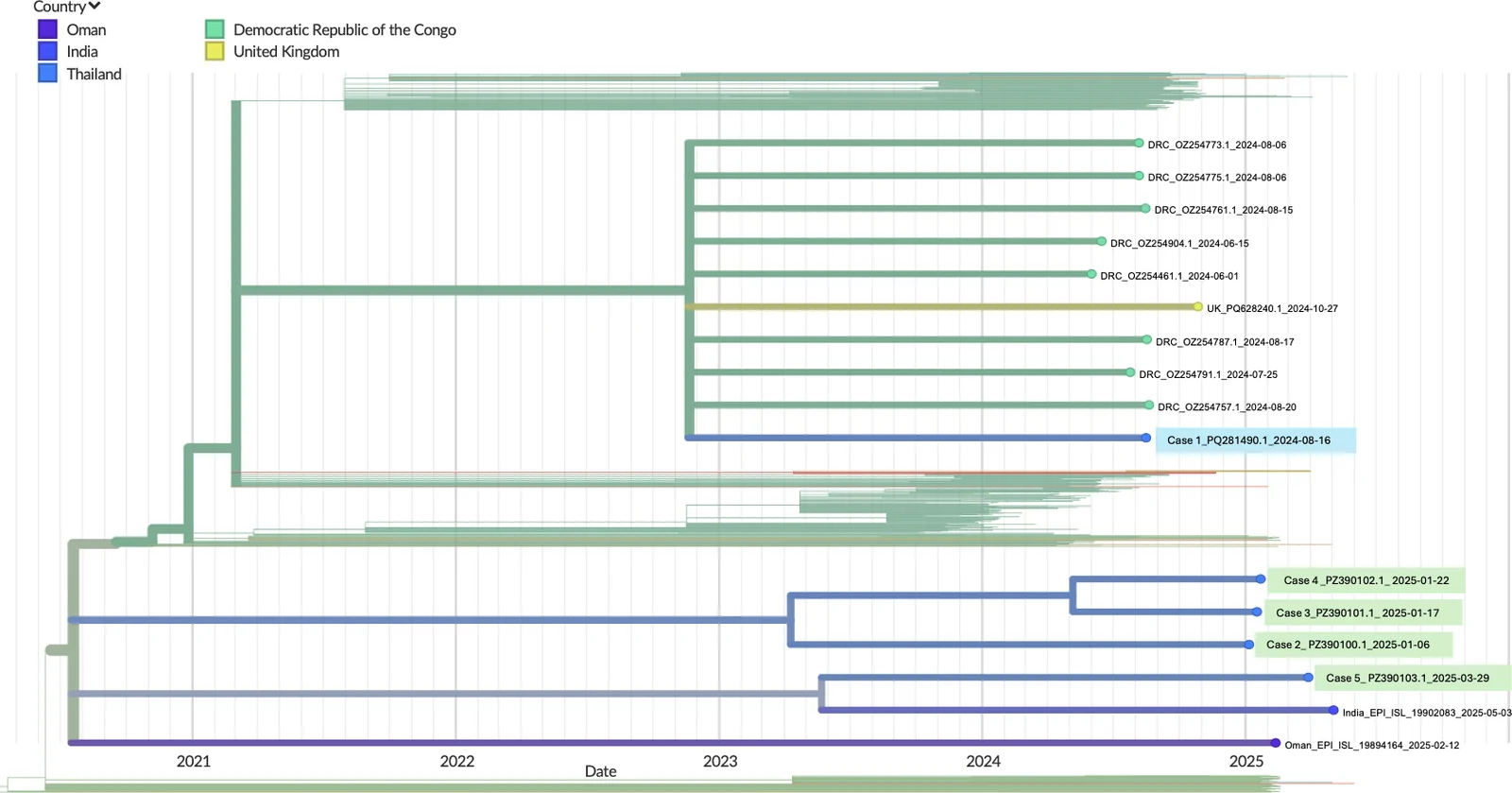
Time-resolved maximum-likelihood phylogeny of MPXV clade Ib genomes from Bamrasnaradura Infectious Diseases Institute (BIDI), Thailand, with 250 contextual global genomes sampled between 2023 and 2025 and retrieved from NCBI and GISAID. Sequence alignment was performed with MAFFT, ML inference with IQ-TREE, and time calibration with TreeTime using the default clade I clock rate, all implemented through the Nextstrain pipeline (v10.3.0) with the MPXV clade I Nextclade data set (tag: 2025-09-09-−12-13-13Z). The *x*-axis represents calendar time (2021–2025) inferred from the molecular clock; tip positions reflect sampling dates. Tip labels are color-coded by country of origin. Among 5 Thai sequences, cases 2–4 form a tight monophyletic cluster with contemporaneous genomes from India and Oman, consistent with a common-source introduction from the United Arab Emirates in late December 2024–January 2025. Case 5 shares a recent common ancestor with Cases 2–4 but is temporally separated (March 2025), supporting an independent second introduction. Case 1 (August 2024) groups separately within DRC-linked diversity, indicating a third independent introduction. Branch support for the corresponding non-time-calibrated topology is provided in Fig. S1.

To resolve the introduction dates of the Thai cases and place them within the global clade Ib phylogeny, we constructed a time-resolved maximum-likelihood (ML) tree. The contextual data set was restricted to clade Ib genomes sampled between 2023 and 2025 (*n*=250 retrieved from NCBI and GISAID on 1 October 2025) because tip-dating relies on contemporaneous sampling within a single evolutionary regime. Clade Ib was first detected in 2023, and no earlier clade Ib genomes exist; inclusion of pre-2023 clade Ia genomes in the time-calibrated tree would bridge two distinct evolutionary regimes (sustained human-to-human clade Ib transmission vs zoonotic clade Ia spillover) and would degrade tip-date resolution. Broader clade Ia/Ib comparisons are presented in a supplementary maximum-likelihood phylogeny (Fig. S1) that includes the clade Ia reference genome DQ011155.1 (Zaire_1979) as an outgroup. Phylogenetic reconstruction was performed with Nextstrain (v10.3.0) using the MPXV clade I Nextclade data set (tag: 2025-09-09-−12-13-13Z). Sequence alignment was performed with MAFFT, which is deterministic and produces a single alignment output; maximum-likelihood inference was performed with IQ-TREE, and time calibration with TreeTime, using the default clade I clock rate and masking scheme. Node dates and 95% confidence intervals were inferred under these defaults. No sequences were collapsed or pruned. Unlabeled tips in Fig. 1 correspond to the 250 contextual global genomes, whose individual labels are omitted for visual clarity; every tip represents a single genome included in the analysis. The resulting JSON output was visualized with Auspice (v2.66.0). To independently confirm clade assignment and topological support, we constructed a supplementary maximum-likelihood phylogeny (Fig. S1) from the same Nextclade alignment, comprising 255 clade Ib genomes (5 Thai and 250 contextual) plus DQ011155.1 as an outgroup. The best-fit substitution model, HKY + F + R3, was selected by ModelFinder under the Bayesian Information Criterion. Branch support was assessed with 1,000 ultrafast bootstrap (UFBoot) and 1,000 SH-aLRT replicates. The tree was rooted on DQ011155.1; clade Ib formed a monophyletic clade with UFBoot = 100, and the five Thai cases occupied the same relative positions as in Fig. 1.

## RESULTS

### Demographic and epidemiological data

The analysis examined five cases of MPXV clade Ib (Table 1). The patients’ ages ranged from 37 to 66 years old. All cases reported histories of intimate or sexual contact. Travel histories included the Democratic Republic of the Congo (DRC) and the United Arab Emirates (UAE). Cases 2, 3, 4, and 5 had been working in Dubai, UAE, before traveling on a direct flight to Thailand. Cases 2 and 3 were found to be friends. All patients presented with symptomatic MPX, including the presence of skin lesions, with lesion counts ranging from 10 to 80 lesions. Accordingly, lesion swab specimens were collected during the symptomatic phase of infection for diagnostic testing.

**TABLE 1:** Demographic, clinical, and epidemiological characteristics of patients infected with MPXV clade Ib in Thailand, August 2024–March 2025

Characteristics	Case 1	Case 2	Case 3	Case 4	Case 5
Age, years	66	39	44	37	45
Sex	Male	Female	Female	Female	Female
Sexual orientation	Heterosexual	Heterosexual	Heterosexual	Heterosexual	Heterosexual
Race and ethnicity	White	Asian	Asian	Asian	Asian
Self-reported contact with a person with suspected MPX	Intimate or sexual contact	Intimate or sexual contact	Intimate or sexual contact	Intimate or sexual contact	Intimate or sexual contact
Travel history	DRC	UAE	UAE	UAE	UAE
Onset date	15 August 24	24 December 24	11 January 25	12 January 25	19 March 25
Symptomatic	Yes	Yes	Yes	Yes	Yes
Presence of skin lesion	Yes	Yes	Yes	Yes	Yes
No. of skin lesions	26–100	80	30	20–30	10–30
Specimen collection date	21 August 24	6 January 25	17 January 25	22 January 25	29 March 25
Specimen types	Lesion swab	Lesion swab	Lesion swab	Lesion swab	Lesion swab
Smallpox vaccination history	No	No	Unknown	No	No
HIV status	Negative	Unknown	Negative	Unknown	Negative
Comorbidities	None	None	None	None	None

### Laboratory findings

The laboratory results presented in Table 2 show that all five confirmed cases tested positive for MPXV, with varying cycle threshold values in real-time PCR testing. The Monkeypox Virus and Central/West African Clade Typing assay detected the presence of MPXV in all five cases but did not distinguish between MPXV clade I and clade II. Case 1 was tested using the QIAstat-Dx Viral Vesicular Panel Assay Version 1, which yielded a positive result for MPXV clade II, as this version was designed to detect orthopoxvirus and MPXV clade II using the same channel. In contrast, Case 2 was analyzed with the QIAstat-Dx Viral Vesicular Panel Assay Version 2, which detected an orthopoxvirus but without clade assignment. The MPXV clade Ib-specific RT-PCR confirmed the presence of MPXV clade Ib in Cases 1, 3, 4, and 5, whereas Case 2 could not be analyzed due to insufficient sample. Whole-genome sequencing confirmed the presence of MPXV clade Ib in all five cases. For Case 1, short-read sequencing using the Illumina Virus Surveillance Panel achieved 99.1% genome coverage with a sequencing depth of 1,008×. For Cases 2–5, combined Illumina and Oxford Nanopore sequencing data were used to generate hybrid genome assemblies, resulting in complete genome coverage (100%) for all four cases. Sequencing depths ranged from 996× to 3,640×, providing high-confidence consensus genomes for downstream phylogenetic and comparative genomic analyses.

**TABLE 2:** Molecular results for MPXV clade Ib cases with specimens collected August 2024–January 2025 in Thailand^*a*^

Lab testing	Case 1	Case 2	Case 3	Case 4	Case 5
1. Real-time PCR					
1.1 Monkeypox Virus and Central/West African Clade Typing					
MPXV	+ Ct = 17.77	+ Ct = 18.10	+ Ct = 14.40	+ Ct = 14.49	+ Ct = 19.10
MPXV clade I	–	–	–	–	–
MPXV clade II	–	–	–	–	–
*1.2 Viral vesicular panel*					
Enterovirus	–	–	NT	NT	–
Herpes simplex virus 1	–	–	NT	NT	–
Herpes simplex virus 2	–	–	NT	NT	–
Human herpes virus 6	–	–	NT	NT	–
Orthopoxvirus+ MPXV clade I^*b*^	–	NT	NT	NT	NT
Orthopoxvirus + MPXV clade II^*b*^	+ Ct = 21.4	NT	NT	NT	NT
Orthopoxvirus^*c*^	NT	+ Ct = 19.50	NT	NT	+ Ct = 19.80
MPXV clade I^*c*^	NT	–	NT	NT	–
MPXV clade II^*c*^	NT	–	NT	NT	–
1.3 MPXV clade Ib (dD14–16)	+ Ct = 19.09	NT	+ Ct = 16.03	+ Ct = 19.48	+ Ct = 21.84
2. Whole-genome sequencing					
Illumina MiniSeq	Clade Ib	Clade Ib	Clade Ib	Clade Ib	Clade Ib
Oxford nanopore	NT	Clade Ib	Clade Ib	Clade Ib	Clade Ib
Bioinformatic tool	Geneious Prime v.2024.0.7	minimap2/ samtools/ bcftools	minimap2/ samtools/ bcftools	minimap2/ samtools/ bcftools	minimap2/ samtools/ bcftools
Length	197,116 bp	195,645 bp	195,628 bp	195,628 bp	195,659 bp
Coverage	99.1%	100%	100%	100%	100%
Depth^*d*^	1,008.0×	966×	1,431×	3,640×	1,178×

^*a*^ + = detected, − = not detected, NT = not tested; Ct values are shown in parentheses.

^*b*^ QIAstat-Dx Viral Vesicular Panel Assay Version 1.

^*c*^ QIAstat-Dx Viral Vesicular Panel Assay Version 2.

^*d*^ Depth values for Cases 2–5 reflect merged Illumina and ONT alignments.

### Time-resolved phylogenetic tree

The tree (Fig. 1) includes global contextual genomes sampled between 2023 and 2025 and colored by country. Among Thai sequences, Cases 2–4 form a tight monophyletic cluster with near-contemporaneous sampling (late December 2024–January 2025), consistent with a single common-source introduction. Case 5 shares a recent common ancestor with Cases 2–4 but is temporally (March 2025) and genetically separated, supporting a second, independent introduction from the UAE. Case 1 (11) groups separately with DRC-linked diversity, indicating a third, independent introduction. Together, the phylogeny indicates ≥3 introductions into Thailand (DRC → Case 1; UAE → Cases 2–4; UAE → Case 5) rather than sustained local transmission since December 2024.

### Genomic analysis

**Fig 2:**
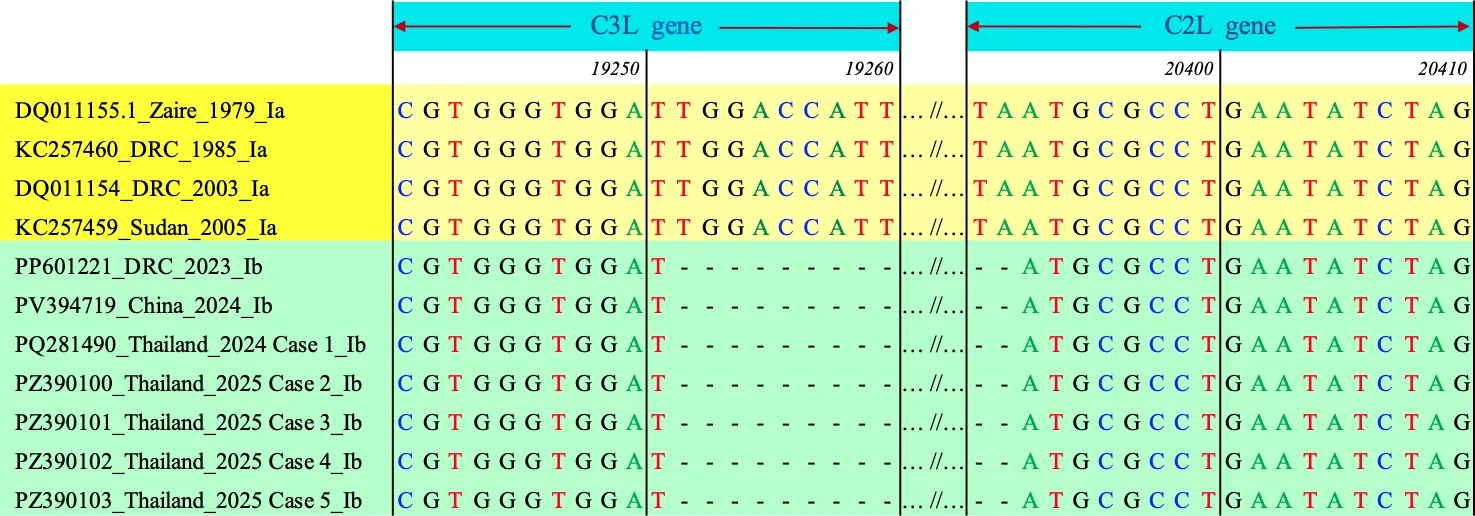
Multiple sequence alignment of MPXV clade Ia and clade Ib sequences reveals a deletion event in the *C3L* gene region of the clade Ib sequences.

Comparative sequence analysis of clade Ia and clade Ib reveals a 1,140 bp deletion in the *C3L* gene region of clade Ib sequences (Fig. 2). This deletion is evident when aligning the sequences with the missing regions highlighted to emphasize their absence in clade Ib. The observed deletion was supported by both Illumina and ONT sequencing data. The commercial assay used in this study targets the *F3L* gene for MPXV detection and the *C3L* gene for clade Ia differentiation. This genomic feature affects the performance of commercial real-time PCR detection kits, which are designed for clade Ia and rely on the targeted amplification of specific genomic regions.

**Fig 3:**
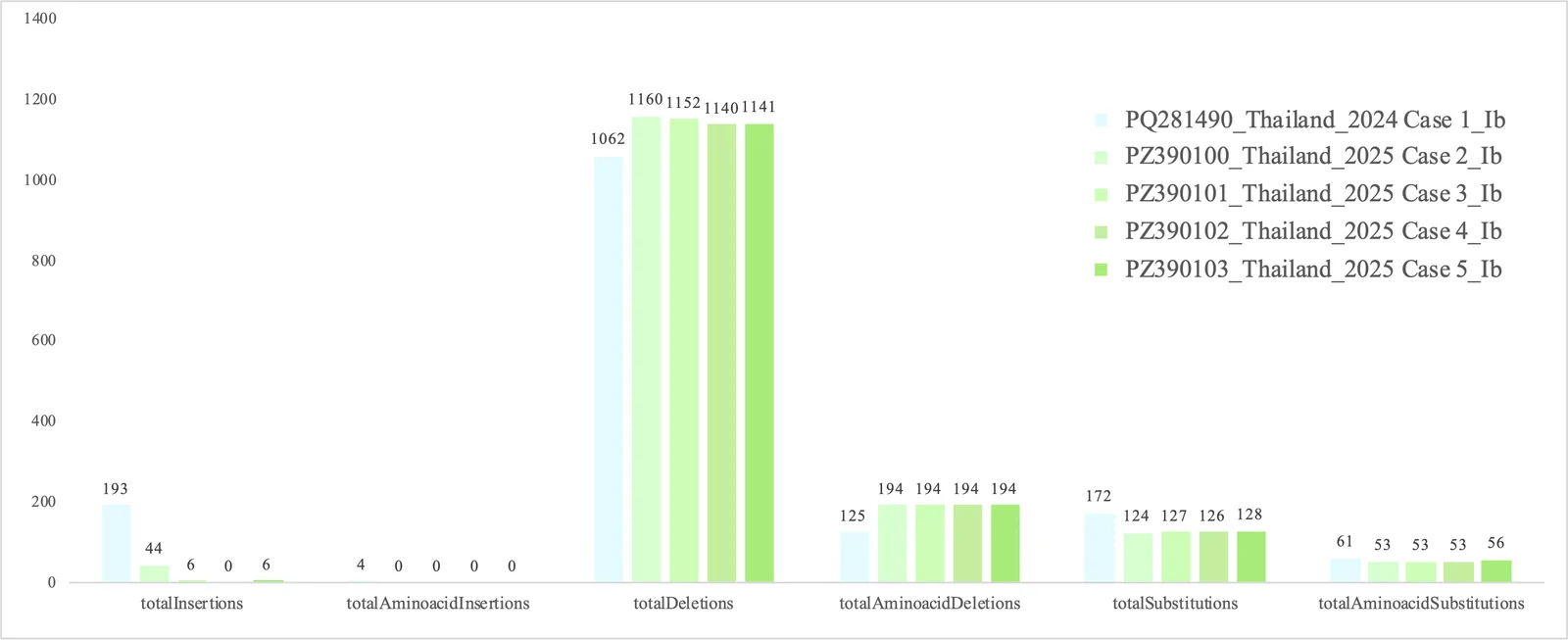
Genomic variation profiles of five Thai MPXV clade Ib genomes compared with the MPXV Clade I reference genome Zaire_1979-005 (GenBank accession number DQ011155.1). Variations were identified using Nextclade v3.21.2 and include total nucleotide insertions, amino acid insertions, nucleotide deletions, amino acid deletions, nucleotide substitutions, and amino acid substitutions relative to the reference genome. Numbers above the bars indicate the total count of each mutation type for each isolate.

**Fig 4:**
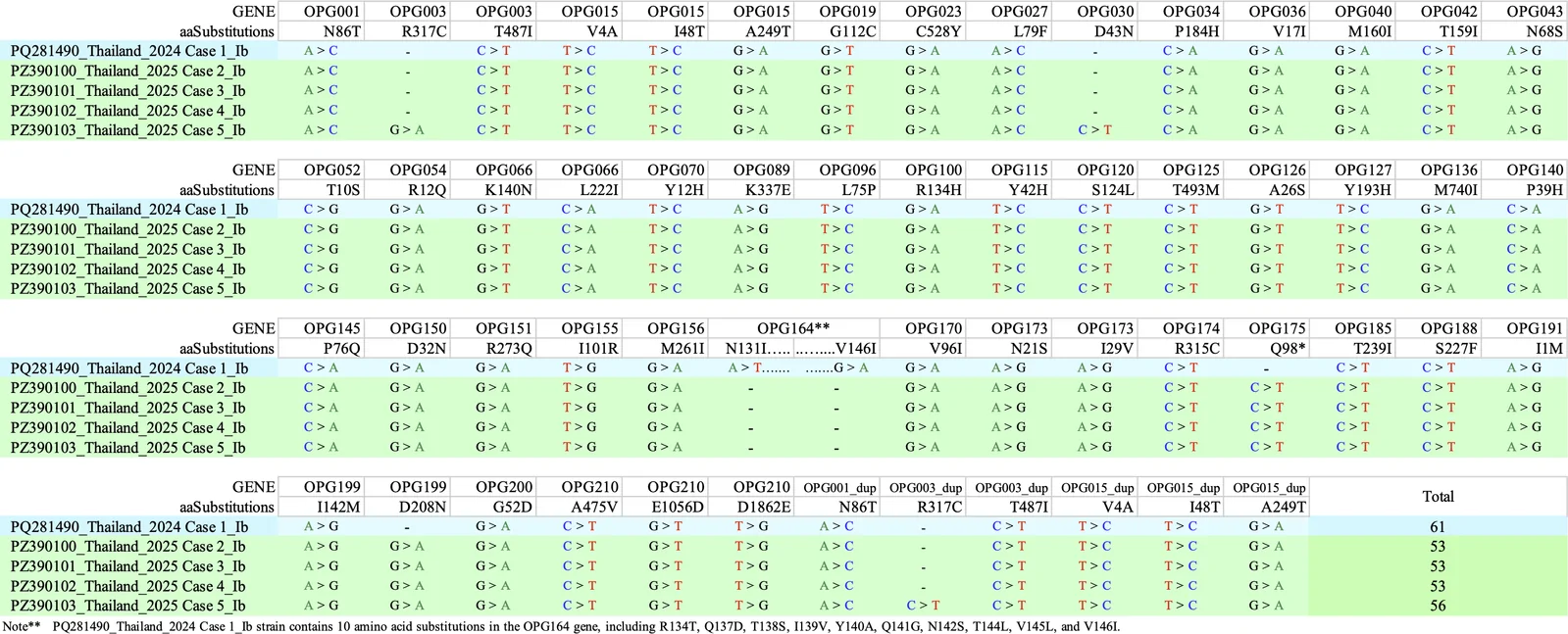
Amino acid substitutions identified in five Thai MPXV clade Ib genomes relative to the MPXV Clade I reference genome Zaire_1979-005 (GenBank accession number DQ011155.1). Amino acid changes were determined using Nextclade v3.21.2. The number of amino acid substitutions per genome ranged from 53 to 61. Case 1 exhibited a distinct substitution pattern in OPG164, whereas Case 5 contained four unique substitutions in OPG003, OPG003_dup, and OPG030.

Using Nextclade v3.21.2 with the Mpox virus (Clade I) data set and the MPXV Clade I reference genome Zaire_1979-005 (GenBank accession number DQ011155.1), we identified nucleotide and amino acid variations in the Thai MPXV clade Ib genomes. Compared with the clade Ia reference genome, the clade Ib genomes exhibited a range of nucleotide and amino acid mutations, including insertions, deletions, and substitutions. These genomic features are summarized in Fig. 3, and the specific amino acid substitutions are detailed in Fig. 4. Among the five cases, Case 1 showed the highest number of nucleotide and amino acid variations, whereas Cases 2–5 displayed highly similar mutation profiles. In Case 1, unique amino acid substitutions were identified in the OPG164 gene (R134T, Q137D, T138S, I139V, Y140A, Q141G, N142S, T144L, V145L, and V146I), while mutations in OPG175 (Q98) and OPG199 (D208N), observed in the other cases, were absent. In addition, four amino acid substitutions in OPG003 (R317C), OPG003_dup (R317C), and OPG030 (D43N) were uniquely detected in the most recent isolate, Case 5.

## DISCUSSION

In August 2024, the first confirmed MPXV clade Ib case was detected in Thailand, making it the second case reported outside of Africa following the clade Ib case detected in Sweden. The first case identified outside of Africa was a Swedish traveler returning in August 2024 from an area in Central Africa affected by the current clade I outbreak (11). Since 2022, the current global MPX outbreak has been driven by multiple sub-lineages within the clade IIb lineage (18) although clade I (Ia/Ib) has continued to cause outbreaks in parts of Central Africa, particularly the Democratic Republic of the Congo (19). To date, clade IIb remains the predominant epidemic clade, affecting primarily men who have sex with men through human-to-human transmission in countries outside of the African continent (20). In Thailand, all identified MPXV clade Ib cases occurred in heterosexual individuals and were linked to intimate or sexual contact.

Following the identification of Case 1, we found a real-time PCR commercial kit for MPXV clade I could not reliably detect MPXV clade Ib. Initial detection of MPXV clade I via real-time PCR was difficult due to the deletion in the *C3L* gene, which was the target for amplification in commercial real-time PCR kits available at the time of testing. The *C3L* gene is often used as a target gene in PCR assays to distinguish between clade I and clade II of MPXV, as the *C3L* gene is completely absent in clade II of MPXV (21–23).

After we discovered that the QIAstat-Dx viral vesicular panel assay kit could not differentiate between MPXV clade II and orthopoxvirus, we communicated our findings to Qiagen Global, and as a result, they revised the kit version 2 to detect MPXV clade Ia and clade II separately. However, at the time of testing, we utilized a clade Ib-specific real-time PCR assay, which enabled rapid and accurate detection and confirmation of MPXV clade Ib. This underscored the pressing need to develop robust diagnostic methods capable of swiftly and precisely identifying MPXV and distinguishing among its various clades and subclades.

Since 2024, travel-associated MPXV clade Ib cases have been reported in multiple countries outside endemic regions, reflecting its international spread. All five cases in this study were associated with travel or exposure outside Thailand, with no evidence of sustained local transmission during the study period. Cases 2, 3, and 4 all reported recent travel from the UAE and developed symptoms within a short interval (late December 2024–January 2025). These cases formed a tight phylogenetic cluster, and the temporal proximity of their onset dates supports the interpretation of a common-source introduction. Case 5, although phylogenetically related and sharing a common ancestor with Cases 2–4, was detected later in March 2025 and showed both temporal and genetic separation from that cluster. Case 1, diagnosed in August 2024, was distinct from both clusters. Together, these data demonstrate that Thailand experienced at least three separate introductions of MPXV clade Ib rather than a single chain of sustained transmission since late 2024.

Our five cases of MPXV clade Ib exhibited a 1,140 bp deletion in the *C3L* gene. C3L, or complement control protein (VCP), effectively prevents the activation of the complement system, which is vital for the innate immune response, thereby aiding in viral evasion (24). The C3L protein was found in both clade I MPXV and the variola virus but was absent in clade II MPXV (25). However, the significance of this deletion remains unclear, and its relationship to disease severity could not be evaluated in this study. Further studies are required to determine its effects on viral fitness, host immune responses, and clinical outcomes.

The genome analysis revealed that the clade Ib genomes exhibit a range of nucleotide and amino acid mutations compared to the clade Ia reference genome, including insertions, deletions, and substitutions. Among the cases, Case 1 had the highest number of these genetic changes, while Cases 2, 3, 4, and 5 showed a similar pattern that aligned with their epidemiological background. The number of amino acid substitutions in the five cases ranged from 53 to 61, comparable to the 57 amino acid mutations observed in the MPXV clade Ib found in China (15). Case 5 exhibited three additional amino acid substitutions on OPG003, OPG030, and the OPG003 duplicate, which were not found in the earlier cases that shared the same origin country and had been identified 2 months prior. Similar genomic changes in orthologous regions were reported during the 2022 outbreak, where multiple substitutions were linked to increased human-to-human transmission and viral evolution under host immune pressure (20, 26).

This study’s main limitation is its small sample size, which constrains broader epidemiological insights. These findings highlight the need for prompt, enhanced case identification, genomic surveillance, expanded epidemiological studies, and continued assessments of vaccine efficacy to address the evolving MPXV clade Ib. Future research should incorporate longitudinal genomic data to better elucidate the evolutionary patterns of MPXV clade Ib.

### Conclusions

The genomic analysis of MPXV clade Ib highlights the need for a coordinated global response. This study identified the first five cases of MPXV clade Ib in Thailand. Key findings include the identification of specific genomic markers, such as a *C3L* gene deletion, and phylogenetic clustering across regions. The identification of this deletion elucidated the limitations of the real-time PCR kit for MPXV clade I, indicating the possible need to develop clade-specific real-time PCR assays for accurately detecting MPXV variants. Future case identification and genomic surveillance will continue to enhance the knowledge base for developing more sensitive and specific assays for the new emerging MPXV clade Ib virus as well. The accumulation of genomic changes in orthologous regions over time suggests a strong link between multiple mutations and increased human-to-human transmission of viruses. Continuous genomic sequencing, coupled with vigilant monitoring of these mutations, is crucial for comprehending viral adaptation and effectively shaping public health strategies.

## Data Availability

The genome sequences generated in this study have been deposited in GenBank under accession numbers PQ281490 and PZ390100–PZ390103. Additional genome sequences and associated metadata used in the analyses are available through GISAID under EPI_SET_260517bv (DOI: 10.55876/gis8.260517bv).

## SUPPLEMENTAL MATERIAL

Fig. S1 spectrum.00516-26-s0001.pdf Comparative alignment of representative historical and contemporary MPXV clade I genomes showing the characteristic 1,140-bp deletion in the *C3L* gene region of clade Ib viruses.

ASM does not own the copyrights to Supplemental Material that may be linked to, or accessed through, an article. The authors have granted ASM a non-exclusive, world-wide license to publish the Supplemental Material files. Please contact the corresponding author directly for reuse.

## References

[1] Giorgi FM, Pozzobon D, Di Meglio A, Mercatelli D. 2024. Genomic and transcriptomic analysis of the recent Mpox outbreak. Vaccine 42:1841–1849. doi:10.1016/j.vaccine.2023.12.086 38311533

[2] Haller SL, Peng C, McFadden G, Rothenburg S. 2014. Poxviruses and the evolution of host range and virulence. Infect Genet Evol 21:15–40. doi:10.1016/j.meegid.2013.10.014 PMC3945082 24161410

[3] Brown K, Leggat PA. 2016. Human monkeypox: current state of knowledge and implications for the future. Trop Med Infect Dis 1:8. doi:10.3390/tropicalmed1010008 PMC6082047 30270859

[4] Kinganda-Lusamaki E, Amuri-Aziza A, Fernandez-Nuñez N, Makangara-Cigolo J-C, Pratt C, Vakaniaki EH, Hoff NA, Luakanda-Ndelemo G, Akil-Bandali P, Nundu SS, et al.. 2025. Clade I mpox virus genomic diversity in the Democratic Republic of the Congo, 2018-2024: predominance of zoonotic transmission. Cell 188:4–14. doi:10.1016/j.cell.2024.10.017 39454573

[5] Chadaga K, Prabhu S, Sampathila N, Nireshwalya S, Katta SS, Tan R-S, Acharya UR. 2023. Application of artificial intelligence techniques for monkeypox: a systematic review. Diagnostics (Basel) 13:824. doi:10.3390/diagnostics13050824 PMC10000611 36899968

[6] Thieme AH, Zheng Y, Machiraju G, Sadee C, Mittermaier M, Gertler M, Salinas JL, Srinivasan K, Gyawali P, Carrillo-Perez F, Capodici A, Uhlig M, Habenicht D, Löser A, Kohler M, Schuessler M, Kaul D, Gollrad J, Ma J, Lippert C, Billick K, Bogoch I, Hernandez-Boussard T, Geldsetzer P, Gevaert O. 2023. A deep-learning algorithm to classify skin lesions from mpox virus infection. Nat Med 29:738–747. doi:10.1038/s41591-023-02225-7 PMC10033450 36864252

[7] Karagoz A, Tombuloglu H, Alsaeed M, Tombuloglu G, AlRubaish AA, Mahmoud A, Smajlović S, Ćordić S, Rabaan AA, Alsuhaimi E. 2023. Monkeypox (Mpox) virus: classification, origin, transmission, genome organization, antiviral drugs, and molecular diagnosis. J Infect Public Health 16:531–541. doi:10.1016/j.jiph.2023.02.003 PMC9908738 36801633

[8] Shchelkunov SN, Totmenin AV, Babkin IV, Safronov PF, Ryazankina OI, Petrov NA, Gutorov VV, Uvarova EA, Mikheev MV, Sisler JR, Esposito JJ, Jahrling PB, Moss B, Sandakhchiev LS. 2001. Human monkeypox and smallpox viruses: genomic comparison. FEBS Lett 509:66–70. doi:10.1016/s0014-5793(01)03144-1 PMC9533818 11734207

[9] World Health Organization. 2024. Mpox global strategic preparedness and response plan. Internet. World Health Organization

[10] Levy V, Branzuela A, Hsieh K, Getabecha S, Berumen R 3rd, Saadeh K, Snyder RE, Marek G, Dodson D, Newman A, Hacker JK, Kath C, Minhaj FS, Gigante CM, Gearhart S, Kallen A, Hutson CL, Jacobson K, Clade I Mpox Response Team. 2025. First clade Ib monkeypox virus infection reported in the Americas - California, November 2024. MMWR Morb Mortal Wkly Rep 74:44–49. doi:10.15585/mmwr.mm7404a1 PMC11824946 39946302

[11] Treutiger C-J, Filén F, Rehn M, Aarum J, Jacks A, Gisslén M, Sturegård E, Karlberg ML, Karlsson Lindsjö O, Sondén K. 2024. First case of mpox with monkeypox virus clade Ib outside Africa in a returning traveller, Sweden, August 2024: public health measures. Euro Surveill 29:2400740. doi:10.2807/1560-7917.ES.2024.29.48.2400740 PMC11605805 39611206

[12] Iacobucci G. 2024. UK reports two further cases of clade Ib mpox. British Medical Journal Publishing Group. 10.1136/bmj.q2442 39500522

[13] de Jong R, Schauer J, Kossow A, Scharkus S, Jurke A. 2024. Response of the German public health service to the first imported mpox clade Ib case in Germany, October 2024. Euro Surveill 29:2400743. doi:10.2807/1560-7917.ES.2024.29.28.2400743 PMC11605798 39611210

[14] Shete AM, Chenayil S, Sahay RR, Sindhu CB, Yadav S, Gawande P, Patil DY, Kumar A, Mohandas S, Yadav PD. 2024. Genomic analysis confirmed the importation of first Mpox clade Ib case in Kerala, India from Dubai, UAE. J Infect 89:106342. doi:10.1016/j.jinf.2024.106342 39505069

[15] Song Y, Yan Y, Xu J, Lv S, Ren G, Zhou Y, Song W, Ge R, Xu P, Zhu G, Chen Z. 2025. Complete genome sequence analysis of the first imported Mpox virus clade Ib variant in China. Pathogens 14:102. doi:10.3390/pathogens14010102 PMC11768240 39861063

[16] Alvi MI, Kliner M, Welfare W, Gordon NC, Thomas S, Padfield S, Emmett HE, Heinsbroek E, Hughes GJ, Groves N, Gallagher E, Pullan S, Belfield A, Houlihan CF, Rampling T, O’Hara G, Tunbridge A, Dunning J, Whittaker E, Alonso A, Beadsworth M, Payne BA, Chand M, Hopkins S, Armstrong G. 2025. Case series of the first five human infections with monkeypox virus clade Ib and report on the public health response, United Kingdom, October to November 2024. Euro Surveill 30:2500131. doi:10.2807/1560-7917.ES.2025.30.10.2500131 PMC11912141 40084422

[17] Schuele L, Masirika LM, Udahemuka JC, Siangoli FB, Mbiribindi JB, Ndishimye P, Aarestrup FM, Koopmans M, Oude Munnink BB, Molenkamp R, GREATLIFE MPOX group, Collaborators. 2024. Real-time PCR assay to detect the novel clade Ib monkeypox virus, September 2023 to May 2024. Euro Surveill 29:2400486. doi:10.2807/1560-7917.ES.2024.29.32.2400486 PMC11312019 39119722

[18] Gao L, Shi Q, Dong X, Wang M, Liu Z, Li Z. 2023. Mpox, caused by the MPXV of the clade IIb lineage, goes global. Trop Med Infect Dis 8:76. doi:10.3390/tropicalmed8020076 PMC9966881 36828492

[19] Vakaniaki EH, Kacita C, Kinganda-Lusamaki E, O’Toole Á, Wawina-Bokalanga T, Mukadi-Bamuleka D, Amuri-Aziza A, Malyamungu-Bubala N, Mweshi-Kumbana F, Mutimbwa-Mambo L, et al.. 2024. Sustained human outbreak of a new MPXV clade I lineage in eastern Democratic Republic of the Congo. Nat Med 30:2791–2795. doi:10.1038/s41591-024-03130-3 PMC11485229 38871006

[20] Isidro J, Borges V, Pinto M, Sobral D, Santos JD, Nunes A, Mixão V, Ferreira R, Santos D, Duarte S, Vieira L, Borrego MJ, Núncio S, de Carvalho IL, Pelerito A, Cordeiro R, Gomes JP. 2022. Phylogenomic characterization and signs of microevolution in the 2022 multi-country outbreak of monkeypox virus. Nat Med 28:1569–1572. doi:10.1038/s41591-022-01907-y PMC9388373 35750157

[21] Saijo M, Ami Y, Suzaki Y, Nagata N, Iwata N, Hasegawa H, Ogata M, Fukushi S, Mizutani T, Iizuka I, Sakai K, Sata T, Kurata T, Kurane I, Morikawa S. 2008. Diagnosis and assessment of monkeypox virus (MPXV) infection by quantitative PCR assay: differentiation of Congo Basin and West African MPXV strains. Jpn J Infect Dis 61:140–142. doi:10.7883/yoken.JJID.2008.140 18362406

[22] Ghate SD, Suravajhala P, Patil P, Vangala RK, Shetty P, Rao RSP. 2023. Molecular detection of monkeypox and related viruses: challenges and opportunities. Virus Genes 59:343–350. doi:10.1007/s11262-023-01975-3 PMC9901828 36746846

[23] Li Y, Zhao H, Wilkins K, Hughes C, Damon IK. 2010. Real-time PCR assays for the specific detection of monkeypox virus West African and Congo Basin strain DNA. J Virol Methods 169:223–227. doi:10.1016/j.jviromet.2010.07.012 PMC9628942 20643162

[24] Ye Q, Zhang D, Zhang R-R, Xu Q, Huang X-Y, Huang B, Sun M-X, Cong Z, Zhu L, Ma J, et al.. 2024. A penta-component mpox mRNA vaccine induces protective immunity in nonhuman primates. Nat Commun 15:10611. doi:10.1038/s41467-024-54909-4 PMC11621575 39639037

[25] Chen N, Li G, Liszewski MK, Atkinson JP, Jahrling PB, Feng Z, Schriewer J, Buck C, Wang C, Lefkowitz EJ, Esposito JJ, Harms T, Damon IK, Roper RL, Upton C, Buller RML. 2005. Virulence differences between monkeypox virus isolates from West Africa and the Congo basin. Virology 340:46–63. doi:10.1016/j.virol.2005.05.030 PMC9534023 16023693

[26] Gigante CM, Korber B, Seabolt MH, Wilkins K, Davidson W, Rao AK, Zhao H, Smith TG, Hughes CM, Minhaj F, et al.. 2022. Multiple lineages of monkeypox virus detected in the United States, 2021-2022. Science 378:560–565. doi:10.1126/science.add4153 PMC10258808 36264825

