# Supplementary material for: Genomic characteristics of the first five monkeypox virus clade Ib cases in Thailand: August 2024–March 2025
Source: Microbiol Spectr. 2026 Sep 24;14(11):e00516-26. doi: 10.1128/spectrum.00516-26 (PMC13729184; doi:10.1128/spectrum.00516-26)
Supplement: Supplementary file 1 [file spectrum.00516-26-s0001.pdf]

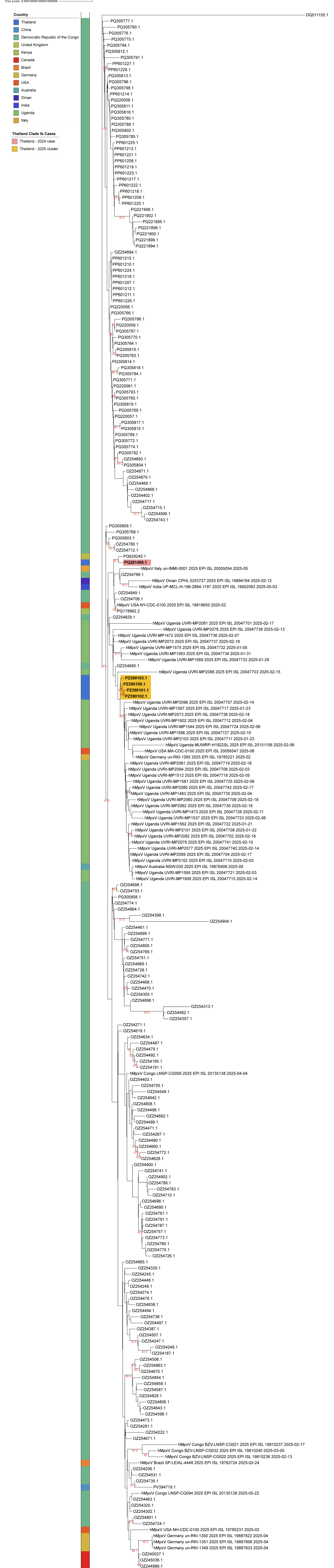
Tree scale: 0.00010000100001000009
Country
Thailand
China
Democratic Republic of the Congo
United Kingdom
Kenya
Canada
Brazil
Germany
USA
Australia
Oman
India
Uganda
Italy
Thailand Clade Ib Cases
Thailand - 2024 case
Thailand - 2025 cluster
DQ011155.1
PQ281490.1
PZ390103.1
PZ390100.1
PZ390101.1
PZ390102.1

Fig S1. Supplementary maximum-likelihood phylogeny of MPXV clade Ib genomes with the clade Ia reference genome DQ011155.1 (Zaire_1979) as an outgroup. The tree was inferred from 256 sequences (255 clade Ib genomes plus DQ011155.1) using the same Nextclade alignment as Figure 1 (MPXV clade I dataset, tag: 2025-09-09--12-13-13Z). The best-fit substitution model (HKY+F+R3) was selected by ModelFinder under the Bayesian Information Criterion, and branch support was assessed with 1,000 ultrafast bootstrap (UFBoot) and 1,000 SH-aLRT replicates. The tree is rooted on DQ011155.1. Internal nodes with UFBoot ≥ 70 are indicated. Country-level phylogeographic bands are displayed in front of the tree, color-coded by sampling location. The five Thai cases are labeled as in Figure 1 and highlighted by year of collection (Case 1 in August 2024; Cases 2–5 in December 2024–March 2025). The Thai cases occupy the same relative phylogenetic positions as in the time-resolved tree (Figure 1), confirming the robustness of the topological inferences and transmission interpretation across methods.
